# DEVELOPING THE CHINESE VERSION OF THE ASSESSMENT OF INTERPROFESSIONAL TEAM COLLABORATION SCALE IN TAIWAN

**DOI:** 10.1093/geroni/igae098.3189

**Published:** 2024-12-31

**Authors:** Hsiao-Wei Yu, Carole Orchard, Yu-Ching Hu

**Affiliations:** National Taiwan University, Taiwan (Republic of China); University of Western Ontario, London, Ontario, Canada; Chang Gung University of Science and Technology, Taoyuan City, Taoyuan, Taiwan (Republic of China)

## Abstract

The delivery of long-term care reablement services emphasizes an interprofessional collaboration (IPC) approach. This study aims to use the existing Assessment of Interprofessional Team Collaboration Scale (AITCS) as an IPC measurement tool, assessing its alignment with Taiwan’s unique caring culture compared to its original use in Canada. Brislin’s Back-Translation Method was employed to create Chinese versions of the AITCS questionnaires, which were then administered to 201 professionals and paraprofessionals involved in reablement services in Taiwan to evaluate reliability and validity. Consistent with the original AITCS, the Chinese Version scale comprises three primary domains—partnership, cooperation, and coordination—with 23 question items. It demonstrates satisfactory reliability (Cronbach’s alpha=0.97 for total scale, ICC=0.91 for test-retest reliability) and acceptable convergent validity (AVE=0.68-0.80, CR>0.90), along with discriminant validity (coefficients < 0.80 between domains). Analysis of IPC performance among participants shows that collaboration (3.82 ± 0.75) and partnership (3.78 ± 0.77) exhibit more favorable scales compared to cooperation (3.56 ± 0.63) (p<.001). This study has preliminarily developed a Chinese Version of the IPC measurement tool, suggesting the need for further investigation into strategies for enhancing cooperation in IPC performance within the context of reablement services in Taiwan.

